# Osteomalacia as a Late Metabolic Complication of Ifosfamide Chemotherapy in Young Adults: Illustrative Cases and Review of the Literature

**DOI:** 10.1155/2007/91586

**Published:** 2007-05-24

**Authors:** D. N. Church, A. B. Hassan, S. J. Harper, C. J. Wakeley, C. G. A. Price

**Affiliations:** ^1^Department of Medical Oncology, Bristol Haematology and Oncology Centre, Horfield Road, Bristol BS2 8ED, UK; ^2^Department of Cellular & Molecular Medicine, School of Medical Sciences, University of Bristol, Bristol BS8 1TD, UK; ^3^Richard Bright Kidney Unit, Southmead Hospital, Westbury-on-Trym, Bristol BS10 5NB, UK; ^4^Department of Radiology, Bristol Royal Infirmary, Marlborough Street, Bristol BS2 8HW, UK

## Abstract

*Purpose*. Ifosfamide is a drug commonly used in the management of sarcomas and other solid tumours. One potential toxicity of its use is renal tubular damage, which can lead to skeletal abnormalities; rickets in children and osteomalacia in adults. We aimed to characterise this rare complication in adults. *Patients*. Three illustrative patient cases treated in our institution are presented. All were treated for sarcoma, and received varying doses of ifosfamide during their therapy. *Methods*. We performed a review of the literature on the renal tubular and skeletal complications of ifosfamide in adults. Papers were identified by searches of PubMed using the terms “osteomalacia,” “nephrotoxicity,” “Fanconi syndrome,” “ifosfamide,” and “chemotherapy” for articles published between 1970 and 2006. Additional papers were identified from review of references of relevant articles. *Results*. There are only four case reports of skeletal toxicity secondary to ifosfamide in adults; the majority of data refer to children. Risk factors for development of renal tubular dysfunction and osteodystrophy include platinum chemotherapy, increasing cumulative ifosfamide dose, and reduced nephron mass. The natural history of ifosfamide-induced renal damage is variable, dysfunction may not become apparent until some months after treatment, and may improve or worsen with time. *Discussion*. Ifosfamide-induced osteomalacia is seldom described in adults. Clinicians should be vigilant for its development, as timely intervention may minimise complications.

## 1. INTRODUCTION

Ifosfamide is an alkylating oxazaphosphorine active against a number of malignancies including bone and soft tissue sarcomas as a single agent [1, 2] or in combination [3]. The development of mesna in 1979 largely ameliorated haemorrhagic cystitis as a complication of its use [4, 5] and the principal associated toxicities became myelosuppression, neurotoxicity, and nephrotoxicity; including glomerular, proximal, and distal tubular dysfunction [6, 7]. If severe, proximal tubular dysfunction may lead to Fanconi syndrome; characterised by glycosuria, aminoaciduria, and excess urinary excretion of phosphate, calcium, bicarbonate, potassium, sodium, and magnesium [8]. Left untreated, hypophosphatemia results in skeletal abnormalities: rickets in children and osteomalacia in adults [8]. While the renal and skeletal consequences of ifosfamide have been well described in the paediatric population [6, 7], there is limited information on such effects in adults.

Here, we report three adult patients in whom osteomalacia as a late consequence of ifosfamide-induced renal tubular dysfunction mimicked skeletal metastases. These cases were diagnosed over a three-year period at a regional cancer centre in which approximately 30–40 patients per year receive ifosfamide-based chemotherapy for sarcoma. In each case osteomalacia was diagnosed on the basis of radiological findings in combination with serum biochemical abnormalities. We define Fanconi syndrome as an acquired renal tubular defect resulting in excess loss of glucose, bicarbonate, amino acids, uric acid, and phosphate. All patients described gave informed consent for publication.

## 2. METHODS

Literature review was performed by searches of PubMed using the terms “osteomalacia,” “nephrotoxicity,” “Fanconi syndrome,” “ifosfamide,” and “chemotherapy” for articles published between 1970 and 2006. Additional papers were identified from the references of relevant articles.

## 3. PATIENTS

### 3.1. Case 1

A 25-year-old man was referred to our institution with an undifferentiated high-grade soft tissue sarcoma of the right thigh in March 1998. He had no history of renal disease and serum electrolytes and creatinine 91 *μ*mol/L were normal at presentation. He received neoadjuvant chemotherapy as described in Table 1 (Case 1, Regimen 1), without complication and with normal serum biochemistry throughout. The tumour responded to treatment and the patient proceeded to radiotherapy (kidneys not included in treatment field) and surgery.

In January 2000, lung metastases were detected in followup imaging and subsequently resected. No further systemic therapy was given. However, in August 2000 unresectable disease progression necessitated palliative chemotherapy (Table 1, Regimen 2). Biochemistry remained within normal range during treatment, and a partial response with effective palliation was achieved.

In June 2001, the patient developed further symptomatic progression of disease. Serum creatinine measured 123 *μ*mol/L, corresponding to a calculated glomerular filtration rate (GFR) of 80ml/min according to the method of Cockroft and Gault [9]. Further chemotherapy was given (Table 1), but discontinued after 4 cycles due to lack of response. In view of the limited treatment options available and their previous efficacy, single-agent ifosfamide was used (Table 1). The patient required hospitalization after the first cycle for management of febrile neutropenia with antibiotics including gentamicin (4 doses at 4 mg/kg daily dose). During this admission transient hypokalemia (nadir 2.6 mmol/L) and elevation of serum creatinine (maximum 134 *μ*mol/L) were observed. Both normalised with supplementation and rehydration and remained within normal range during subsequent courses. Treatment resulted in significant tumour regression and effective palliation.

**Figure 1:**
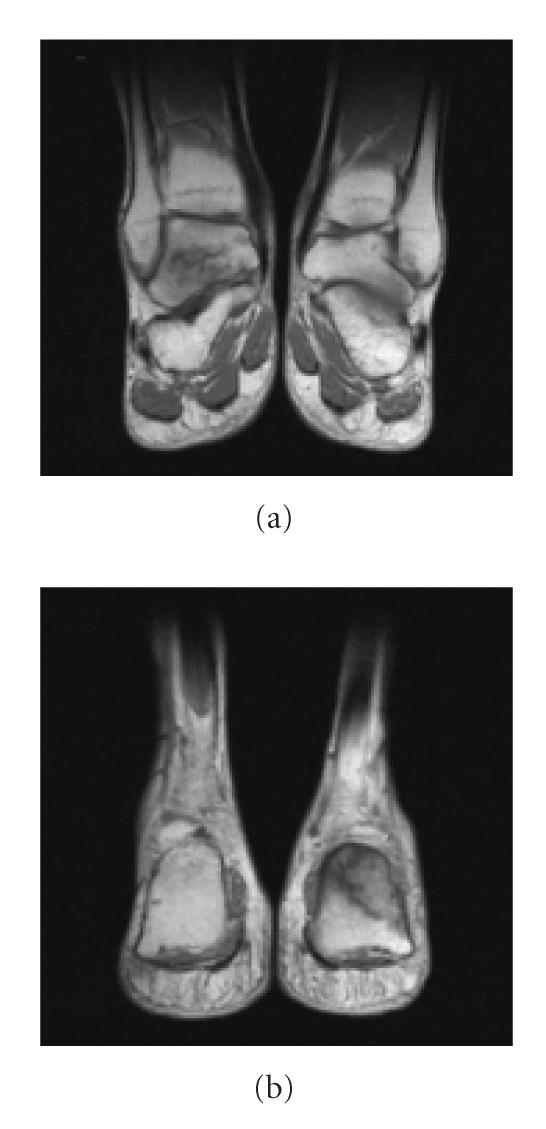
Case 1: T1-weighted magnetic resonance (MR) demonstrating stress fractures of (a) right talus and (b) left calcaneum.

In April 2002, five months after cessation of ifosfamide treatment, concomitant with further progression of pulmonary disease, the patient complained of bilateral ankle pain following a snowboarding holiday. Isotope bone scan demonstrated increased uptake at sites of symptoms and serum alkaline phosphatase measured 266 IU/L. Though the possibility of skeletal metastases was considered, careful review of plain radiographs revealed insufficiency fractures of both ankles, confirmed on magnetic resonance (MR) scan (Figure 1). Biochemistry confirmed the characteristic hypokalaemia and hypophosphataemia (0.8 mmol/L) of renal Fanconi syndrome. There was no urinary sediment and serum whole parathyroid hormone (PTH) was normal. Oral alfacalcidol and potassium supplementation were prescribed.

Unfortunately, over the following months a steady decline in glomerular function developed with haemodialysis initiated in February 2003. During this period, continued disease progression occurred and despite treatment with two nonnephrotoxic regimens (etoposide and gemcitabine) the patient died from disseminated malignancy in August 2003.

### 3.2. Case 2

**Figure 2:**
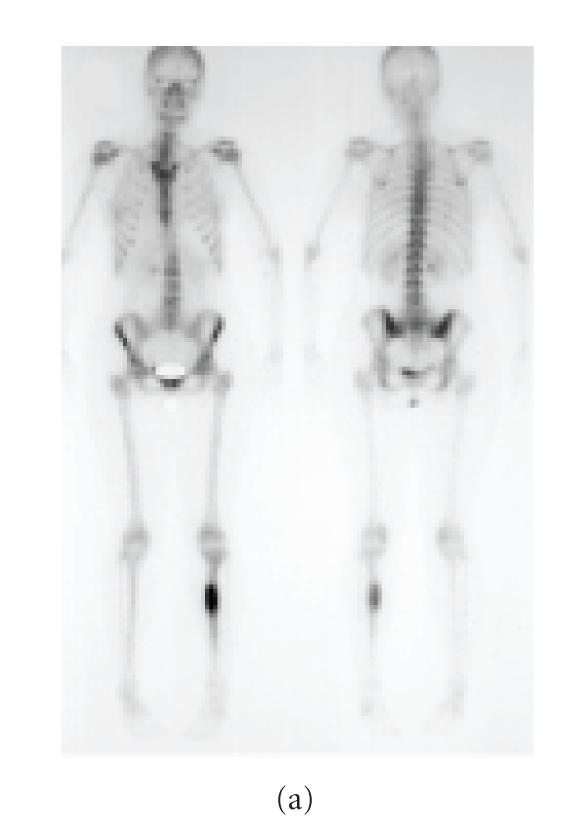

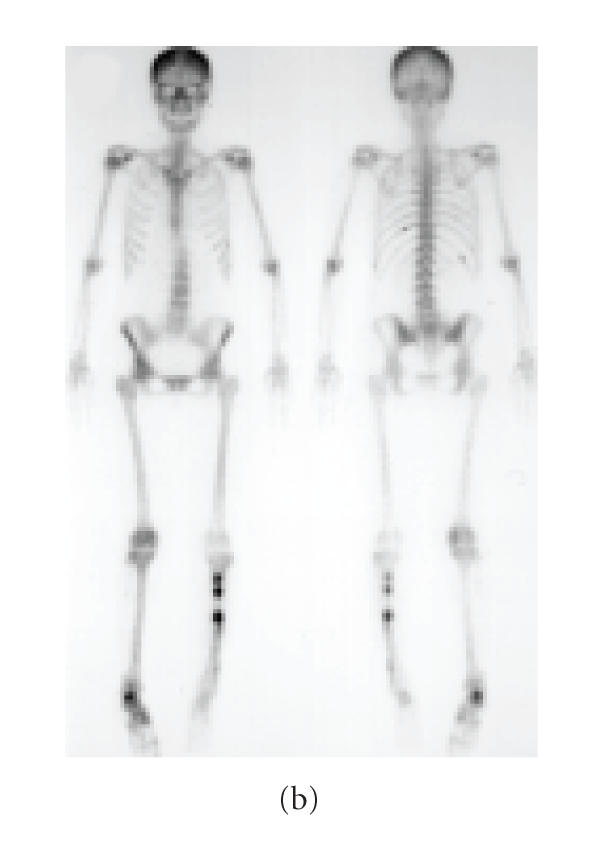
Case 2: isotope bone scans, (a) at diagnosis, demonstrating increased uptake at site of primary tumor in left tibia, (b) after ifosfamide therapy and surgery, demonstrating continued increased uptake at site of primary tumor and new abnormality in region of right ankle.

A 33-year-old woman with Ewing's sarcoma of the left tibia was referred for management in May 2002. She had no history of renal disease, and serum creatinine at presentation measured 98 *μ*mol/L. Initial isotope bone scan is shown in Figure 2(a).

Neoadjuvant chemotherapy was administered, using the VIDE regimen [10] (Table 1). After the fifth cycle, the patient was hospitalised for management of febrile neutropenia, including 6 doses of gentamicin at 4 mg/kg daily dose (levels within normal range throughout). During this admission transient hypokalemia (nadir 3.1 mmol/L) and modest elevation of serum creatinine 115 *μ*mmol/L occurred, though both normalised with supplementation and rehydration. In view of this toxicity, the sixth cycle was prescribed at reduced dose, but further hypokalaemia (nadir 2.7 mmol/L) necessitated transient oral supplementation. After completion of chemotherapy, the patient underwent tumour excision with a free fibula graft.

Following surgery, adjuvant therapy was commenced in December 2002 as per standard practice in our institution. One cycle of VAI [10] (Table 1) was given, but complicated by febrile neutropenia requiring hospitalization for treatment including 3 doses of gentamicin at 4 mg/kg. During this admission an asymptomatic rise in creatinine to 167 *μ*mol/L occurred. Cyclophosphamide was therefore substituted for ifosfamide for subsequent courses, which were well tolerated with biochemistry remaining within normal range throughout.

Six months after completion of therapy and 10 months after surgery, the patient still complained of significant pain at the operation site. Plain radiographs demonstrated nonunion of the fibula graft but also areas of lucency in the proximal and distal tibia are demonstrated. Isotope bone scan added to suspicion of metastases, with several areas of increased uptake evident (Figure 2(b)). MR performed to further delineate the abnormality revealed a stress fracture within the fibula graft, and biopsies confirmed healing tissue only with no evidence of malignancy. Serum biochemistry demonstrated hypokalaemia and elevated alkaline phosphatase, confirming the diagnosis of osteomalacia secondary to Fanconi syndrome. No urinary sediment was identified, serum whole PTH was normal throughout. Oral alfacalcidol supplementation was commenced.

As of May 2006, the patient is clinically well under joint oncology and nephrology followup with no evidence of recurrent malignancy. She continues to require vitamin D supplementation and serum creatinine remains elevated, but stable at 133 *μ*mol/L.

### 3.3. Case 3

A 31-year-old man was referred in May 2004 for further management of a peripheral neuroectodermal tumour of the left kidney, having undergone left nephrectomy the preceding month. He had no history of renal disease and serum creatinine at presentation measured 81 *μ*mol/L.

Staging investigations demonstrated no evidence of metastases and he therefore received adjuvant therapy using the VIDE regimen [10] (Table 1). Following the second cycle of treatment, the patient required hospitalization for management of nausea and vomiting. Significant biochemical derangement was noted on admission; serum creatinine measured 224 *μ*mol/L; urea 11.1 mmol/L; potassium 2.9 mmol/L; phosphate <0.3 mmol/L, and corrected calcium 2.06 mmol/L. Electrolytes normalised with supplementation and serum creatinine fell to 129 *μ*mol/L prior to cycle 3, for which a reduced dose of ifosfamide was used. Despite this, the patient again required hospital admission for management of febrile neutropenia after the two subsequent cycles, on both occasions treated without aminoglycosides. Although serum potassium remained within normal range during this period without supplementation, following the fourth and final cycle marked hypophosphatemia (nadir <0.3 mmol/L) and elevation of serum creatinine 162 *μ*mol/L were noted. Treatment was completed in September 2004.

During initial followup serum creatinine remained elevated but stable at 143–157 *μ*mol/L and the patient was able to discontinue all electrolyte supplementations. However in November 2004 a progressive decline in glomerular function became evident, with increase in creatinine to 300 *μ*mol/L. To exclude obstructive uropathy secondary to retroperitoneal tumour recurrence as cause of this, a CT of the abdomen and pelvis was performed. While this demonstrated no evidence of outflow obstruction, it revealed a number of abnormal areas throughout the skeleton. A bone scan to further assess these showed increased uptake in the region of the left ankle. Further biochemical analysis confirmed elevated alkaline phosphatase, hypocalcaemia, and hypophosphataemia; consistent with osteomalacia secondary to Fanconi syndrome. Whole PTH was normal with no urinary sediment. Oral alfacalcidol supplementation was commenced.

Unfortunately, glomerular function continued to decline over subsequent months, renal biopsy revealed marked interstitial fibrosis, with dialysis initiated in August 2005. At time of this report (May 2006), the patient is free from recurrence, under joint oncology and nephrology followup.

## 4. DISCUSSION

The three cases presented illustrate the potential for the delayed development of osteomalacia secondary to ifosfamide-induced Fanconi syndrome in adult patients. Cumulative drug doses were variable; 110 g/m^2^, 57 g/m^2^, and 24 g/m^2^ in Cases 1, 2, and 3, respectively. In addition to tubular dysfunction, each patient has shown evidence of associated deranged glomerular filtration, with two patients requiring dialysis.

Ifosfamide is associated with a variety of renal toxicities (see Skinner [6, 7] for comprehensive reviews) including glomerular dysfunction leading to fall in glomerular filtration rate (GFR) [11–15], distal tubular damage leading to poor urinary concentrating ability [16, 17], and more commonly, proximal tubular dysfunction [7, 13, 15] thought to be caused by chloroacetaldehyde [18–20], a metabolite of ifosfamide toxic to human proximal tubular cells [21]. Although mesna is theoretically capable of detoxifying chloroacetaldehyde, its efficacy may be incomplete [22] and its rapid tubular excretion may further impair any protective action at this site.

The incidence of proximal tubular dysfunction in children treated with ifosfamide varies according to the criteria used for assessment. While subclinical glycosuria was detected in 88% of patients in a large study [14], clinically significant abnormalities including Fanconi syndrome occur in between 1.3 and 27% of treated patients [11, 17, 23, 24]. Risk is increased by prior or concurrent platinum chemotherapy [11, 13, 15, 25, 26], increasing cumulative ifosfamide dose [12–14, 17, 25, 27], and reduced functioning nephron mass [13, 23, 25, 28], but not scheduling of ifosfamide dose [29, 30]. Whether aminoglycoside use increases risk is unclear [14, 31] although aminoglycosides per se may cause Fanconi syndrome [32].

It is important to note that whilst tubular damage secondary to ifosfamide may become apparent during therapy, it can develop months or even years after cessation of treatment [33, 34] as in these cases. The subsequent natural history is equally unpredictable; though damage may resolve with time [6, 33], with spontaneous resolution of secondary rickets [35], there may be no improvement in tubular function [34] and an associated progressive decline in GFR can occur [23, 36]. End-stage renal failure in adults secondary to ifosfamide has previously been documented in three patients after cumulative ifosfamide doses of 56 g/m^2^, 80 g/m^2^ and 33 g/m^2^ [15]. All of these patients had been pretreated with *cis*platin, whereas only one of the cases we report had previously received this agent.

Hypophosphatemic rickets occurs in 5–18% [17, 23, 24] of children treated with ifosfamide and may be the first manifestation of the underlying renal abnormalities [23, 24]. There are only four published cases of adults with Fanconi syndrome and renal osteomalacia secondary to ifosfamide [37–40]. In three of these, patients have displayed associated symptoms of significant renal dysfunction such as polyuria and polydipsia. By contrast, our patients were clinically well at the time of diagnosis of stress fractures, leading to the suspicion of bone metastases.

Our experience highlights the fact that tubular and skeletal complications of ifosfamide are not restricted exclusively to a high-risk paediatric population. Indeed our second case occurred in a patient without any risk factors receiving standard drug doses. These findings clearly have implications for patient monitoring during and after ifosfamide chemotherapy. Whilst a comprehensive assessment of kidney function as described in the paediatric literature [41] may not be appropriate for all adult patients, assessment of GFR by ^51^Cr-EDTA clearance [42] and tubular function by serum and urine electrolyte measurements (including calculation of the renal tubular threshold for phosphate) [41] is straightforward and may help prevent the complications we describe. Unfortunately, the paucity of data on this subject in adults means that when faced with subtle changes in tubular function, clinicians may not have the evidence available upon which to base decisions as how to adjust ifosfamide doses.

These cases demonstrate the need for clinicians to be vigilant for signs of renal tubular dysfunction in patients receiving ifosfamide, particularly if *cis*platin is pretreated, as timely intervention may prevent skeletal complications of this drug. They also illustrate the point that when faced with a patient in the clinic with bone pain and abnormal isotope bone scan after ifosfamide treatment, oncologists should be mindful that the cause may in fact be insufficiency fractures secondary to osteomalacia rather than metastatic disease, and futher skeletal imaging may be required to differentiate between the two. This clinical caveat may be of increasing relevance, as a recent study has documented hypophosphataemia and abnormal bone metabolism in patients treated with the tyrosine kinase inhibitor imatinib, likely through inhibition of the platelet-derived growth factor (PDGF) receptor [43].

## Figures and Tables

**Table 1:** Chemotherapeutic regimens used in cases. *Cis*: cisplatin, dox: doxorubicin, ifos: ifosfamide, epi: epirubicin, vinb: vinblstine, dac: dacarbazine, vinc: vincristine, etop: etoposide, act: actinomycin D, cyclo: cyclophosphamide. Note that *cis*platin was administered with preand posthydration and ifosfamide was demonstrated as an infusion with concurrent mesna in all regimens.

	Regimen 1	Regimen 2	Regimen 3	Regimen 4	Total ifos dose

	Apr–Jul 1998	Aug–Nov 2000	Jun–Aug 2001	Oct-Nov 2001	
Case 1	*Cis* 100 mg/m^2^, d1 Dox 25 mg/m^2^, d1–3 *Alternating with* Ifos 5 g/m^2^, d1 Dox 25 mg/m^2^, d1–3 *5 cycles total*	Ifos 3.33 g/m^2^, d1–4 *5 cycles (#1 at reduced dose)*	Epi 90 mg/m^2^, d1 *Cis* 75 mg/m^2^, d1 *Alternating with* Vinb 5 mg/m^2^, d1 *Cis* 75 mg/m^2^, d1 Dac 800 mg/m^2^, d1 *4 cycles total*	Ifos 3.33 g/m^2^, d1–4 * 3 cycles*	
Total ifos	10 g/m^2^	60 g/m^2^		40 g/m^2^	110 g/m^2^

	Jun–Oct 2002	Dec 2002–Feb 2003			
Case 2	Vinc 1.4 mg/m^2^, d1 Ifos 3 g/m^2^, d1–3 Dox 20 mg/m^2^, d1–3 Etop 150 mg/m^2^, d1–3^10^ * 6 cycles (#6 at 65% dose)*	Vinc 1 mg/m^2^, d1 Act 0.75 mg/m^2^, d1, 2 Ifos 3 g/m^2^ d1, 2 *3 cycles, cyclo-sub- stituted for ifos after #1*	—	—	
Total ifos	51 g/m^2^	6 g/m^2^	—	—	57 g/m^2^

	Jun–Sept 2004				
Case 3	VIDE (as above)^10^ * 4 cycles (reduced dose ifos from #3)*	—	—	—	
Total ifos	24 g/m^2^	—	—	—	24 g/m^2^

## References

[1] Bramwell VHC, Mouridsen HT, Santoro A (1987). Cyclophosphamide versus ifosfamide: final report of a randomized phase II trial in adult soft tissue sarcomas. *European Journal of Cancer and Clinical Oncology*.

[2] Antman KH, Ryan L, Elias A, Sherman D, Grier HE (1989). Response to ifosfamide and mesna: 124 previously treated patients with metastatic or unresectable sarcoma. *Journal of Clinical Oncology*.

[3] Patel SR, Vadhan-Raj S, Burgess MA (1998). Results of two consecutive trials of dose-intensive chemotherapy with doxorubicin and ifosfamide in patients with sarcomas. *American Journal of Clinical Oncology*.

[4] Bruhl P, Hoefer-Janker H, Scheef W, Vahlensieck W (1979). Prophylactic alkalization of the urine during cytostatic tumor treatment with the oxazaphosphorine derivatives, cyclophosphamide and ifosfamide. * Onkologie*.

[5] Scheef W, Klein HO, Brock N (1979). Controlled clinical studies with an antidote against the urotoxicity of oxazaphosphorines: preliminary results. *Cancer Treatment Reports*.

[6] Skinner R (2003). Chronic ifosfamide nephrotoxicity in children. *Medical and Pediatric Oncology*.

[7] Skinner R, Sharkey IM, Pearson ADJ, Craft AW (1993). Ifosfamide, mesna, and nephrotoxicity in children. *Journal of Clinical Oncology*.

[8] Foreman JW, Roth KS (1989). Human renal Fanconi syndrome—then and now. *Nephron*.

[9] Cockcroft DW, Gault MH (1976). Prediction of creatinine clearance from serum creatinine. *Nephron*.

[10] Strauss SJ, McTiernan A, Driver D (2003). Single center experience of a new intensive induction therapy for ewing's family of tumors: feasibility, toxicity, and stem cell mobilization properties. *Journal of Clinical Oncology*.

[11] Pratt CB, Meyer WH, Jenkins JJ (1991). Ifosfamide, Fanconi's syndrome, and rickets. *Journal of Clinical Oncology*.

[12] Ashraf MS, Brady J, Breatnach F, Deasy PF, O'Meara A (1994). Ifosfamide nephrotoxicity in paediatric cancer patients. *European Journal of Pediatrics*.

[13] Rossi R, Godde A, Kleinebrand A (1994). Unilateral nephrectomy and cisplatin as risk factors of ifosfamide-induced nephrotoxicity: analysis of 120 patients. *Journal of Clinical Oncology*.

[14] Skinner R, Cotterill SJ, Stevens MCG (2000). Risk factors for nephrotoxicity after ifosfamide treatment in children: a UKCCSG Late Effects Group study. United Kingdom Children's Cancer Study Group. *British Journal of Cancer*.

[15] Marinez F, Deray G, Cacoub P, Beaufils H, Jacobs C (1996). Ifosfamide nephrotoxicity: deleterious effect of previous cisplatin administration. *The Lancet*.

[16] Rossi R, Godde A, Kleinebrand A, Rath B, Jurgens H (1995). Concentrating capacity in ifosfamide-induced severe renal dysfunction. *Renal Failure*.

[17] Skinner R, Pearson ADJ, English MW (1996). Risk factors for ifosfamide nephrotoxicity in children. *The Lancet*.

[18] Zamlauski-Tucker MJ, Morris ME, Springate JE (1994). Ifosfamide metabolite chloroacetaldehyde causes Fanconi syndrome in the perfused rat kidney. *Toxicology and Applied Pharmacology*.

[19] Mohrmann M, Ansorge S, Schmich U, Schonfeld B, Brandis M (1994). Toxicity of ifosfamide, cyclophosphamide and their metabolites in renal tubular cells in culture. *Pediatric Nephrology*.

[20] Springate JE (1997). Ifosfamide metabolite chloroacetaldehyde causes renal dysfunction in vivo. *Journal of Applied Toxicology*.

[21] Dubourg L, Michoudet C, Cochat P, Baverel G (2001). Human kidney tubules detoxify chloroacetaldehyde, a presumed nephrotoxic metabolite of ifosfamide. *Journal of the American Society of Nephrology*.

[22] Mohrmann M, Ansorge S, Schonfeld B, Brandis M (1994). Dithio-bis-mercaptoethanesulphonate (DIMESNA) does not prevent cellular damage by metabolites of ifosfamide and cyclophosphamide in LLC-PK_1_ cells. *Pediatric Nephrology*.

[23] Burk CD, Restaino I, Kaplan BS, Meadows AT (1990). Ifosfamide-induced renal tubular dysfunction and rickets in children with Wilms tumor. *Journal of Pediatrics*.

[24] Suarez A, McDowell H, Niaudet P, Comoy E, Flamant F (1991). Long-term follow-up of ifosfamide renal toxicity in children treated for malignant mesenchymal tumors: an International Society of Pediatric Oncology report. *Journal of Clinical Oncology*.

[25] Loebstein R, Koren G (1998). Ifosfamide-induced nephrotoxicity in children: critical review of predictive risk factors. *Pediatrics*.

[26] Marina NM, Poquette CA, Cain AM, Jones D, Pratt CB, Meyer WH (2000). Comparative renal tubular toxicity of chemotherapy regimens including ifosfamide in patients with newly diagnosed sarcomas. *Journal of Pediatric Hematology/Oncology*.

[27] Skinner R, Pearson ADJ, Price L, Coulthard MG, Craft AW (1990). Nephrotoxicity after ifosfamide. *Archives of Disease in Childhood*.

[28] Rossi R, Kleinbrand A, Godde A, Rath B, Jurgens H (1993). Increased risk of ifosfamide-induced renal Fanconi's syndrome after unilateral nephrectomy. *The Lancet*.

[29] Boddy AV, English M, Pearson ADJ, Idle JR, Skinner R (1996). Ifosfamide nephrotoxicity: limited influence of metabolism and mode of administration during repeated therapy in paediatrics. *European Journal of Cancer—Part A*.

[30] Rossi R, Schafers P, Pleyer J, Postler C, Boos J, Jurgens H (1997). The influence of short versus continuous ifosfamide infusion on the development of renal tubular impairment. *International Journal of Pediatric Hematology/Oncology*.

[31] Rossi RM, Kist C, Wurster U, Kulpmann W-R, Ehrich JHH (1994). Estimation of ifosfamide/cisplatinum-induced renal toxicity by urinary protein analysis. *Pediatric Nephrology*.

[32] Melnick JZ, Baum M, Thompson JR (1994). Aminoglycoside-induced Fanconi's syndrome. *American Journal of Kidney Diseases*.

[33] Caron HN, Abeling N, van Gennip A, de Kraker J, Voucte PA (1992). Hyperaminoaciduria identifies patients at risk of developing renal tubular toxicity associated with ifosfamide and platinate containing regimens. *Medical and Pediatric Oncology*.

[34] Rossi R (1997). Nephrotoxicity of ifosfamide—moving towards understanding the molecular mechanisms. *Nephrology Dialysis Transplantation*.

[35] van Gool S, Brock P, Wijndaele G (1992). Reversible hypophosphatemic rickets following ifosfamide treatment. *Medical and Pediatric Oncology*.

[36] Prasad VK, Lewis IJ, Aparicio SR (1996). Progressive glomerular toxicity of ifosfamide in children. *Medical and Pediatric Oncology*.

[37] Garcia AA (1995). Ifosfamide-induced Fanconi syndrome. *Annals of Pharmacotherapy*.

[38] Negro A, Regolisti G, Perazzoli F, Davoli S, Sani C, Rossi E (1998). Ifosfamide-induced renal Fanconi syndrome with associated nephrogenic diabetes insipidus in an adult patient. *Nephrology Dialysis Transplantation*.

[39] Beckwith C, Flaharty KK, Cheung AK, Beatty PG (1993). Fanconi's syndrome due to ifosfamide. *Bone Marrow Transplantation*.

[40] Duck L, Devogelaer J-P, Persu A (2005). Osteomalacia due to chemotherapy-induced Fanconi syndrome in an adult patient. *Gynecologic Oncology*.

[41] Skinner R, Pearson ADJ, Coulthard MG (1991). Assessment of chemotherpy-associated nephrotoxicity in children with cancer. *Cancer Chemotherapy and Pharmacology*.

[42] Rehling M, Moller ML, Thamdrup B (1984). Simultaneous measurement of renal clearance and plasma clearance of ^99m^Tc-labelled diethylenetriaminepenta-acetate, ^51^Cr-labelled ethylenediaminetetra-acetate and inulin in man. *Clinical Science*.

[43] Berman E, Nicolaides M, Maki RG (2006). Altered bone and mineral metabolism in patients receiving imatinib mesylate. *New England Journal of Medicine*.

